# Large and tunable magnetocaloric effect in gadolinium-organic framework: tuning by solvent exchange

**DOI:** 10.1038/s41598-019-51590-2

**Published:** 2019-10-30

**Authors:** Vladimír Zeleňák, Miroslav Almáši, Adriána Zeleňáková, Pavol Hrubovčák, Róbert Tarasenko, Sandrine Bourelly, Philip Llewellyn

**Affiliations:** 1 Institute of Chemistry, Faculty of Science, P.J. Šafárik University in Košice, Moyzesova 11, SK-041 54, Košice, Slovakia; 2 Institute of Physics, Faculty of Science, P.J. Šafárik University in Košice, Park Angelinum 9, SK-040 01 Košice, Slovakia; 3 Aix-Marseille University, CNRS, MADIREL, F-13397, Marseille 20, France

**Keywords:** Inorganic chemistry, Condensed-matter physics

## Abstract

Magnetic properties of three variants of MOF-76(Gd), {[Gd(BTC)(H_2_O)]·G}_n_ (BTC = benzene-1,3,5-tricarboxylate, G = guest molecules) were investigated by static susceptibility, isothermal magnetization and specific heat capacity measurements. In the study we used as synthesized MOF-76(Gd)-DMF (**1**) (G = DMF = dimethylformamide), containing DMF molecules in the cavity system, compound MOF-76(Gd) (**2**), activated complex without solvents in the cavities and water exchanged sample MOF-76(Gd)-H_2_O (**3**). A pronounced change in the magnetic entropy was found near the critical temperature for all three compounds. It was shown, that magnetic entropy change depends on the solvatation of the MOF. The highest value entropy change, ΔS_Mpk_(T) was observed for compound **2** (ΔS_Mpk_(T) = 42 J kg^−1^ K^−1^ at 1.8 K for ΔH = 5 T). The ΔS_Mpk_(T) for the compounds **1, 2** and **3** reached 81.8, 88.4 and 100% of the theoretical values, respectively. This suggests that in compound **3** Gd^3+^···Gd^3+^ antiferromagnetic interactions are decoupled gradually, and higher fields promote a larger decoupling between the individual spin centers. The observed entropy changes of compounds were comparable with other magnetic refrigerants proposed for low-temperature applications. To study the magnetothermal effect of **2** (the sample with largest −ΔS_Mpk_), the temperature-dependent heat capacities (C) at different fields were measured. The value of magnetic entropy S obtained from heat capacities (39.5 J kg^−1^ K^−1^ at 1.8 K for an applied magnetic field change of 5 T) was in good agreement with that derived from the magnetization data (42 J kg^−1^ K^−1^ at 1.8 K).

## Introduction

Increase in energy demand in almost all areas of human activity necessitates its careful and properly managed use. Therefore, one of the greatest challenges on mankind in the 21^st^ century relies upon the increasing the efficiency in energy utilization. Particularly, energy demand for cooling is the fastest growing and thus his efficiency can save a lot of financial sources. Energy efficient cooling can also contribute to reduction of carbon trace and green gas emissions like CO_2_, since most of electricity is still produced by burning of fossil fuels.

One of the promising technologies, that enable cooling with higher environmental and economic efficiency is technology based on the magnetocaloric effect (MCE). This technique is more advantageous than conventional gas compression (vapor-cycling technologies)^1,2^. MCE is 20–50% more efficient, than conventional gas compression^3^. The MCE can be described as the change of the magnetic entropy and adiabatic temperature in response to a change in the applied magnetic field and can be exploited for magnetic refrigeration in a process known as adiabatic demagnetization^4,5^. The magnitude of the MCE of a magnetic material is characterized by ΔS_M_, the isothermal magnetic entropy change, and ΔT_ad_, the adiabatic temperature change.

From synthetic point of view, one of the effective strategies to design materials with a high MCE is utilization of isotopic Gd^3+^ ion with seven unpaired 4 f electrons and the maximum entropy (109.9 J.kg^−1^.K^−1^). Different kinds of gadolinium-based materials have attracted interest for MCE, including discrete molecules multinuclear metal clusters (molecular nanomagnets) or materials with extended structures (1D → 3D) with ΔS_m_ values from 1.9 to 50.1 J.kg^−1^.K^−1^ under applied field change of 7 T^6–11^. One subgroup of materials with 3D extended structures are metal-organic frameworks (MOFs), also known as porous coordination polymers. These materials perform superior functional properties and application potential in gas storage, separation, heterogeneous catalysis^12–22^, while their magnetocaloric characteristics have rarely been scrutinized^23–25^.

In MOF compounds, the solvatation state can lead to crystal phase transition and can influence the distances between magnetic metal centers, and consequently also their magnetic properties and MCE. Therefore, the aim of our work is to investigate, how the MCE in MOFs can be influenced by the solvent exchange. For the study we have used gadolinium form of metal-organic framework MOF-76^26^. This compound combines the qualities of isolated paramagnetic Gd^3+^ centers and the robust framework with strong covalent bond connections in the three dimensions.

## Methods

All chemicals used in the synthesis were of the highest available purity as purchased from Sigma-Aldrich Company and used without further purification.

### Synthesis

As synthesized sample MOF-76(Gd)-DMF was prepared according to procedure described in^26^. Activated sample, {[Gd(BTC)]}_n_, without DMF and H_2_O solvents in the cavity system was prepared by heating of as synthesized complex at 400 °C in an oven and the sample was denoted as MOF-76(Gd). Water exchanged sample MOF-76(Gd)-H_2_O with formulae {[Gd(BTC)(H_2_O)]·4H_2_O}_n_ was prepared from activated form by its dispersion in distilled water. Elemental and ICP-MS analyses for compounds: MOF-76(Gd)-DMF, Gd_1_C_12_H_12_N_1_O_8_ (455.48 g mol^−1^): calculated: Gd, 34.52%; C, 31.64%; H, 2.66%; N, 3.08%; found: Gd, 34.38%; C, 31.87%; H, 2.56%; N, 3.15%; MOF-76(Gd), Gd_1_C_9_H_3_O_6_ (364.37 g mol^−1^): calculated: Gd, 47.16%; C, 29.67%; H, 0.83%; found: Gd, 47.24%; C, 29.56%; H, 0.87%; MOF-76(Gd)-H_2_O, Gd_1_C_9_H_13_O_11_ (454.44 g mol^−1^): calculated: Gd, 34.60%; C, 23.79%; H, 2.88%; found: Gd, 34.77%; C, 23.58%; H, 2.94%. For detailed information about IR spectra and thermal stability of the samples see Tables S1 and S2, respectively.

### Elemental and ICP-MS analyses

The elemental analysis was performed with CHNOS Elemental Analyzer vario MICRO from Elementar Analysensysteme GmbH with sample weight approximately 2 mg. The amount of gadolinium(III) in prepared compound was determined in atmosphere of argon on ICP-MS 7700 instrument developed by Agilent Technologies.

### Infrared spectroscopy

IR spectra of the compounds were recorded in the form of KBr pellets with complex/KBr mass ratio 1/100 and using a Nicolet 6900 spectrometer in the wavenumber range 4000–400 cm^−1^. Before IR measurements KBr was dried at 700 °C for 3 h in an oven and cooled in desiccator. All spectra were collected with a resolution of 4 cm^−1^ by collecting 32 scans for a single spectrum. The *in-situ* heating IR spectra (HT-IR) were measured using Thermo Smart Proteus equipment in temperature range 25–400 °C with heating rate 5 °C.min^−1^. The spectra were measured with 10 °C step after 1 min. standing of the sample at desired temperature.

### Thermogravimetry (TG)

The TG/DTG (DTG = derivate thermogravimetry) and DTA (differential thermal analysis) measurements were carried out using the Netsch 409 PC instrument under dynamic conditions with a heating rate of 10 °C.min^−1^. The mass of the samples was within the 20–30 mg. The samples have been heated under air atmosphere with flow rate 20 cm^3^.min^−1^ in the temperature range from 30 to 900 °C.

### High-energy powder X-ray diffraction (HEPXRD)

HEPXRD measurements were carried out at BW5 wiggler beamline of DORIS positron storage ring in DESY (Deutches Elektronen Synchrotron, Hamburg, Germany). The spectra were measured at room temperature and during *in-situ* heating (HT-HEPXRD) up to 600 °C under the conditions as described in^27^.

### Magnetic susceptibility measurements

Temperature dependence of molar susceptibility was obtained by SQUID based magnetometer MPMS 5XL (Quantum Design) in the temperature range 1.8–300 K on three samples MOF-76(Gd)-DMF, MOF-76(Gd) and MOF-76(Gd)-H_2_O in both regimes ZFC/FC (zero field cooled and field cooled regimes) in magnetic field 100 mT. The powder specimens (20.6 mg for MOF-76(Gd)-DMF, 20.2 mg for MOF-76(Gd), and 17.8 mg for MOF-76(Gd)-H_2_O) were fixed in a gelatine capsule and the capsule was held by a straw. The signal contribution of empty gel cap and the straw was subtracted from the total signal. Also, the obtained data were corrected for the diamagnetic contribution using Pascal’s constants.

### Heat capacity measurements

The specific heat capacity was measured using the option in PPMS (Quantum Design) at the constant pressure, under external magnetic fields 0–9 T and in conventional temperature range 2–55 K using ^4^He refrigerator and in the temperature range 0.4–2 K using ^3^He insert. The contribution of the addenda was measured and subtracted from the total specific heat data. The powder samples were fixed to a sample platform using grease Appiezon N.

## Results and Discussion

### Structure, morphology and mechanical properties

**Figure 1:**
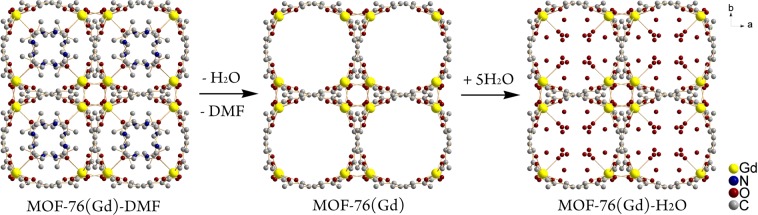
A view of the structures of MOF-76(Gd)-X samples and solvent exchange process (X = DMF – compound **1**, X = no solvent – compound **2**, or X = H_2_O – compound **3**). Hydrogen atoms are omitted for clarity purposes.

Microporous isostructural lanthanide-based MOFs of composition {[Ln(BTC)(H_2_O)]·G}_n_ or MOF-76(Ln) (Ln = lanthanide, BTC = benzene-1,3,5-tricarboxylate, G = guest molecules)^12–25^ represent a class of 3D transformable frameworks, which exhibit permanent porosity and high thermal stability. In the study we used three compounds: compound **1 -** as synthesized MOF-76(Gd)-DMF (DMF = dimethylformamide), containing DMF molecules in the cavity system; compound **2** - MOF-76(Gd) activated complex without solvents in the cavities; and compound **3 -** water exchanged sample MOF-76(Gd)-H_2_O. As synthesized sample MOF-76(Gd)-DMF, i.e. sample with the formulae {[Gd(BTC)(H_2_O)]·DMF}_n_, was prepared according to procedure described in^26^. The desolvated sample MOF-76(Gd), sample with the formulae {[Gd(BTC)]}_n_, was obtained after heating of MOF-76(Gd)-DMF at 400 °C in oven for 3 hrs. MOF-76(Gd)-H_2_O was prepared from MOF-76(Gd) by its dispersion in distilled water. Water exchanged sample MOF-76(Gd)-H_2_O with formulae {[Gd(BTC)(H_2_O)]·4H_2_O}_n_ was prepared from MOF-76(Gd) by its dispersion in distilled water. The scheme of the preparation of the compounds **1–3** and the view of their crystal structures along *c* crystallographic axis is shown in Fig. 1.

The crystal structures of MOF-76(Gd)-X (X = DMF – compound **1**, no solvent – compound **2**, or H_2_O – compound **3**) are built from Gd(III) ions connected *via* carboxylate groups of BTC^3−^ linker giving of one-dimensional left handed helical chains propagating along *c* axis. In the 1D chains, each Gd^3+^ ion has two neighboring Gd^3+^ ions at the distances of 4.718 Å for MOF-76(Gd)-DMF, 4.296 Å for MOF-76(Gd) and 4.326 Å for MOF-76(Gd)-H_2_O. Connection of Gd(III) and BTC^3−^ led to formation of a 3D rod-packing framework with 1D sinusoidally-shaped channels of size about 6.6 × 6.6 Å^2^ along the *c* axis. The Gd^3+^….Gd^3+^ distances between Gd^3+^ ions in neighboring 1D chains are much longer, being in the range 8–11 Å. In the prepared compounds, the cavity system is filled with DMF/water as guest molecules (sample MOF-76(Gd)-DMF, compound **1**), water (sample MOF-76(Gd)-H_2_O, compound **2**) or cavity system is empty (sample MOF-76(Gd)) (see Fig. 1).

Solvent removal/exchange and thermal stability of the samples were studied by combination of thermogravimetric analysis (TGA, Fig. S1 and Table S1 in ESI), infrared spectroscopy of the samples pre-heated to different temperatures (FT-IR, Fig. S2 and Table S2 in ESI), and high-energy powder X-ray diffraction measured during *in-situ* heating (HEPXRD, Fig. S3 in ESI). Based on TGA it was shown, that the desolvatation process in the samples MOF-76(Gd)-DMF and MOF-76(Gd)-H_2_O takes place in temperature range 80–400 °C upon removal solvents from the structure cavities and water molecule coordinated to Gd^3+^ ion. For the sample MOF-76(Gd)-DMF, i.e. {[Gd(BTC)(H_2_O)]·DMF}_n_, the removal of 1H_2_O and 1DMF molecule was accompanied by mass loss 20.89% (calcd. 20.00%) and for the sample MOF-76(Gd)-H_2_O, i.e. {[Gd(BTC)(H_2_O)]·4H_2_O}_n_ the release of 5 moles 5H_2_O was accompanied by mass loss 20.18% (calcd. 19.82%). The TG curve of MOF-76(Gd) (see Fig. S1 in ESI) remains unchanged up to 600 °C, indicating that all the solvate molecules, have been removed. The FT-IR spectra of the samples MOF-76(Gd)-DMF and MOF-76(Gd)-H_2_O treated at different conditions also confirm the gradual release of guest molecules, H_2_O and DMF (see Fig. S2 in ESI). HEPXRD pattern of prepared MOF-76(Gd)-DMF measured during *in-situ* heating (Fig. S3 in ESI) shows two distinct structural changes in temperature range 25–600 °C. Starting from the as-synthesized sample the most relevant changes in the patterns were observed at 140 °C and 320 °C. The framework started to transform after heating to 140 °C due to transformation of crystallographic system from tetragonal to monoclinic. Subsequent heating to 320 °C resulted in minor shifts of the peak positions. Upon heating to higher temperatures crystal structure transformed back to tetragonal system. The HEPXRD patterns of the compounds **1–3** confirmed their crystallinity and structural integrity and are displayed as inset of Fig. S3 in ESI. Rietveld analysis of the patterns confirmed small structural changes and showed, that the presence or absence of the solvents in the cavity system have a significant influence on the Gd···Gd distances with the values of 4.718 Å for MOF-76(Gd)-DMF, 4.296 Å for MOF-76(Gd) and 4.326 Å for MOF-76(Gd)-H_2_O.

Porosity and porous structure of sample MOF-76(Gd), obtained after the solvent removal was also studied by CO_2_ and CH_4_ adsorption and measurement of adsorption enthalpies. The difference between the CO_2_ and CH_4_ adsorbed is weak. The energetic profiles during the adsorption on the surface and the filling of the porosity (slightly increasing for CO_2_ and quite constant for CH_4_) reveal an energetically homogeneous surface (see Fig. S4 in ESI).

### Magnetic properties

**Figure 2:**
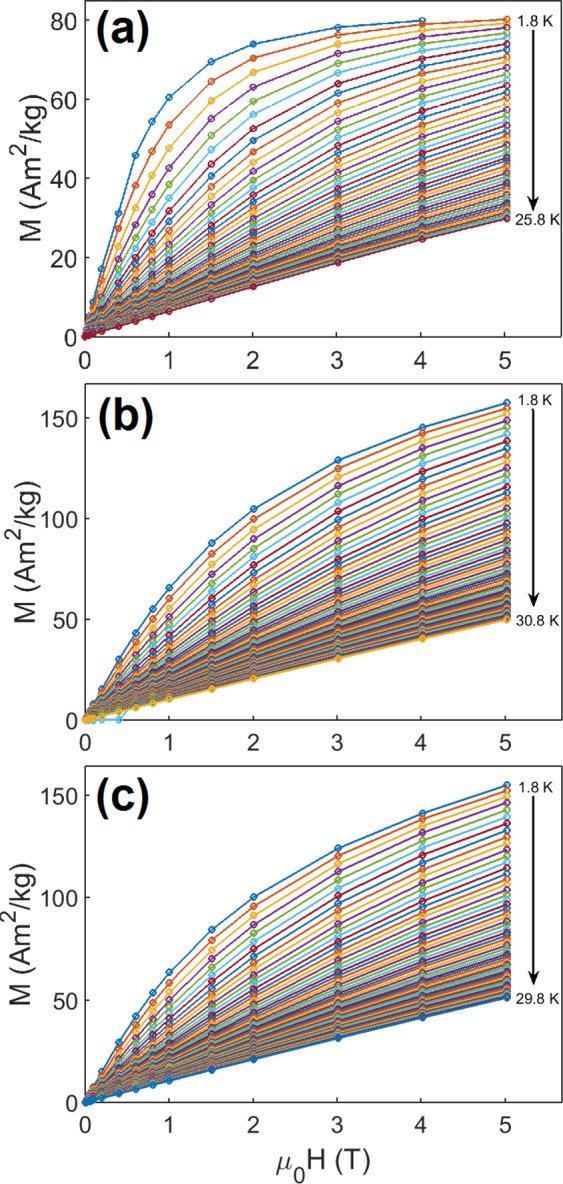
Isothermal magnetization data of studied compounds up to applied field of 5 T obtained at temperature range 1.8 K–29.8 K with the step 1 K for compounds **1** (**a**), **2** (**b**) and **3** (**c**).

The inverse magnetic susceptibility versus temperature (see Fig. S5 in ESI) perfectly follows the Curie–Weiss law and a linear fit for temperature 10–300 K gives values of Θ = 1.54 K for MOF-76(Gd)-DMF, Θ = −6.23 K for MOF-76(Gd), and Θ = −5.58 K for MOF-76(Gd)-H_2_O. No difference between the magnetic response of the field cooled (FC) and zero-field-cooled (ZFC) magnetic regimes in studied compounds was observed, indicating no transition to the long-range ordered state. The very weak ferromagnetic interaction (FM) in compound MOF-76(Gd)-DMF was indicated by a low positive value of Θ. In opposite, the presence of antiferromagnetic exchange interaction (AFM) between Gd^3+^ ions was evidenced from the negative values of Θ in complexes MOF-76(Gd) and MOF-76(Gd)-H_2_O. Besides this, dominant paramagnetic behavior in all studied compounds can be confirmed from temperature and field dependence of magnetization, see Fig. S5 in ESI. The determined magnetic parameters are summarized in Table S3 in ESI. The ferromagnetic behavior in gadolinium based metal–organic framework was observed e.g. in MOF prepared by the reaction of H_2_EDA ligand (H_2_EDA = (ethylenedithio)acetic acid)^28^, where density functional theory calculations showed that the ferromagnetic properties originated from the 4f electrons of Gd(III) propagating by a super-exchange pathway on two −/+/− spin nets of the carboxylate groups^28^. The parameters of the exchange interactions calculated for our three samples **1–3** achieve values of J/k_B_ = 0.3 K for MOF-76(Gd)-DMF, J/k_B_ = −1.8 K for MOF-76(Gd), and J/k_B_ = −1.06 K for MOF-76(Gd)-H_2_O. The values of parameters of the exchange interactions for samples **1–3** sensitively reflect on different distances between Gd···Gd ions in the framework of the samples: 4.718 Å for MOF-76(Gd)-DMF – compound **1**, 4.296 Å for MOF-76(Gd) - compound **2**, and 4.326 Å for MOF-76(Gd)-H_2_O – compound **3**. This suggests, that the solvent exchange, which affects the distance between neighboring Gd ions in the framework, has a significant effect on magnetic properties and thus the possibility of influencing the parameters of the magnetocaloric phenomenon by different solvents can be expected. To evaluate the magnetocaloric effect (MCE), isothermal magnetization from 1.8 to 30 K was measured for the compounds **1**, **2** and **3**, see Fig. 2.

The relationship between magnetization changes and magnetic entropy can be expressed by Maxwell relation *(∂M/∂T)*_*H*_ = *(∂S/∂H)*_*T*_^29^, which for an isothermal-isobaric process after integration yields Eq. 1 ^30,31^ expressed as:

1 $$\Delta {S}_{M}=\int {(\frac{\partial M}{\partial T})}_{H}dH.$$

**Figure 3:**
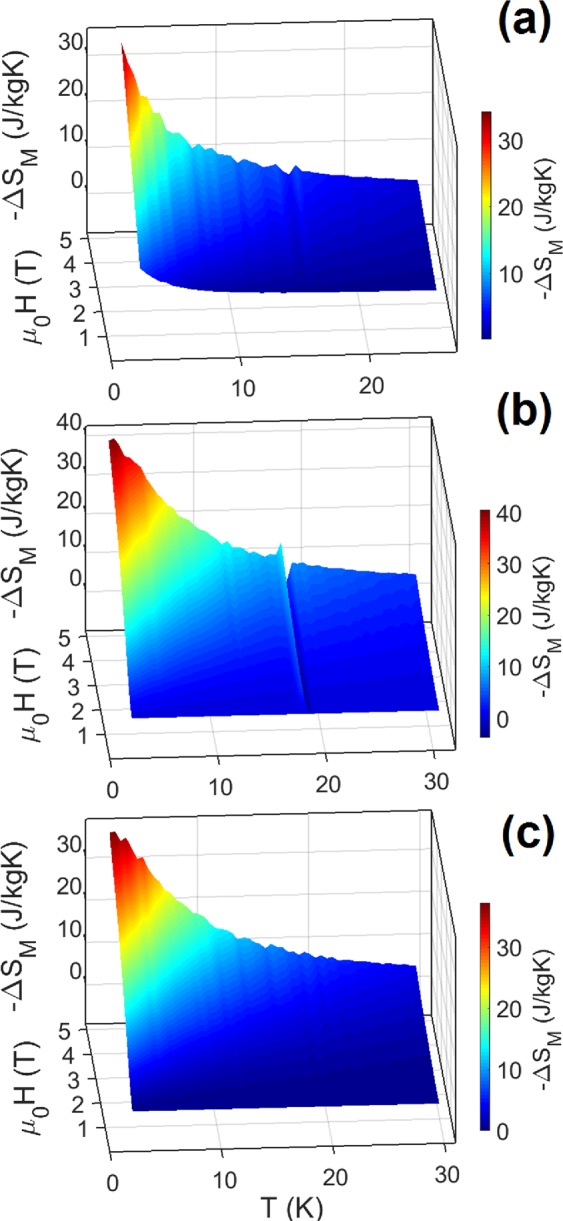
Magnetic entropy change *vs*. temperature of studied compounds up to applied field of 5 T calculated from isothermal magnetization data. 3D view on −ΔS_M_ evolution for **1 (a**), **2** (**b**) and **3** (**c**).

As it is known, the magnetic entropy change −ΔS_M_ can be obtained from the experimental magnetization data at various magnetic fields and temperatures from the Maxwell equation, Eq. 1. The results of −ΔS_M_ calculated from experimental data at different magnetic fields and temperatures are given in Fig. 3. With the aim to estimate the magnetocaloric properties of studied samples 1–3 *via* magnetic entropy change, the isothermal magnetization data recorded by standard measurement protocol was applied. The details of standard protocol are described in ESI.

The −ΔS_M_(T) value shows a gradual increase with increasing ΔH and decreasing temperature, giving −ΔS_Mpk_(T) = 31 J kg^−1^ K^−1^ for MOF-76(Gd)-DMF, −ΔS_Mpk_(T) = 42 J kg^−1^K^−1^ for MOF-76(Gd) and −ΔS_Mpk_(T) = 38 J kg^−1^ K^−1^ for MOF-76(Gd)-H_2_O at an applied field change of 5 T (−ΔS_Mpk_ = peak of magnetic entropy change). At a first sight, the value of −ΔS_Mpk_ changes with the different distances between Gd···Gd ions in the framework of the samples: 4.718 Å for MOF-76(Gd)-DMF, 4.296 Å for MOF-76(Gd) and 4.326 Å for MOF-76(Gd)-H_2_O. However, we can obtain different view based on the formula ΔS_M_ = nRln(2 s + 1)/M_w_ (where R is the gas constant and s represents the effective spin, M_w_ is a molar weight of the sample and *n* is a number of noninteracting spins). The theoretical maximum entropy value per mole, −ΔS_M_, for isotopic Gd^3+^ ion with seven unpaired 4 f electrons and spin *s* = 7/2, the corresponding to n = 1, is 109.9 J.kg^−1^.K^−1^ ^32–35^. For the MOF compounds, where the molar mass is larger due to presence of organic ligands, the maximal theoretical entropy change calculated using this formula is lower. Therefore it is necessary to expect other (lower) ΔS values than calculated according to the above mentioned theoretical equation. Calculated maximal entropy change for the compound **1**, {[Gd(BTC)(H_2_O)]·DMF}_n_, is 37.9 J.kg^−1^.K^−1^, for the compound **2**, {[Gd(BTC)]}_n_, the theoretical value is 47.5 J.kg^−1^.K^−1^ and for the compound **3**, {[Gd(BTC)(H_2_O)]·4H_2_O}_n_, the theoretical value is 38.0 J.kg^−1^.K^−1^. Apparently, in our study the highest value ΔS_Mpk_(T) was observed for compound **2** (ΔS_Mpk_(T) = 42 J kg^−1^ K^−1^ at 1.8 K for ΔH = 5 T). This value does not reach the theoretical one (47.5 J kg^−1^ K^−1^).

In general, the difference between measured and theoretical values mainly originates from the magnetic interactions and crystal-field effects. The coupling between Gd^3+^ ions as well as their finite magnetic anisotropies, reduce the achievable entropy content. The lower experimental values were also observed for compound **1**, but no discrepancy between calculated and measured values was observed in sample **3**. The ΔS_Mpk_(T) for the compounds **1**, **2** and **3** reached 81.8, 88.4 and 100% of the theoretical values, respectively. This suggests that in compound **3** Gd^3+^···Gd^3+^ antiferromagnetic interactions (see Fig. S5 and Table S3 in ESI) are decoupled gradually and higher fields promote a larger decoupling between the individual spin centers, yielding an increasingly much better agreement and entropy to approaches the maximum value for fully decoupled, non-interacting single-ion spins. It should be emphasized that the nature of the interactions (which originate from the Curie Law) between Gd ions in studied MOFs compounds may be antiferromagnetic, but these interactions are upset above the Néel temperature and hence the system is in a paramagnetic state. Therefore no inverse MCE is expected.

The observed values ΔS_Mpk_(T) are comparable to other Gd-MOF compounds. A list of selected 3D MOFs based on gadolinium (III) with magnetic entropy changes at various applied field change magnitudes and temperature 2 K are presented in Table 1. It is to note, that the entropy changes in carboxylate MOFs mainly depends on the position and interconnection of magnetically active centers in final crystal structure, which are modulated by the several factors such as gadolinium distance, bulkiness of the ligand or ion dimensionality (ID). ID expresses the spatial spreading of Gd(III) ions bridged only by carboxylate group of linker. The higher dimensionality usually leads to higher the entropy change (see Tab. 1). Compound {[Gd(HCOO)(BDC)]}_n_ (BDC = terephthalate, FOR = formate = HCOO) is structurally similar to MOF-76(Gd)-X (see Fig. S6 in ESI). The crystal structure of {[Gd(FOR)(BDC)]}_n_ is formed by Gd^3+^ ions bridged by HCOO to form a 2D plains, which are further pillared with BDC linkers. Complex exhibits maximum entropy change of 47 J.kg^−1^.K^−1^ at 2.3 K and ΔH = 9 T^36^. Another comparison can be performed with 3D polymer {[Gd(FOR)_3_]}_n_ in which Gd(III) ions are triangularly arranged by formates in magnetically frustrated lattice^37^. Formate as the smallest and the simplest carboxylate molecule led to formation of framework with 3D ion dimensionality with short Gd···Gd distance of 3.988 Å. The combination of all three aspects short Gd···Gd distance, small linker and high dimensionality of metal centers resulted in the highest entropy change 56 J.kg^−1^.K^−1^ observed for gadolinium-based MOFs. Despite of the fact that MOF-76(Gd) represents material with 1D ion dimensionality, observed entropy change 42 kJ.kg^−1^.K^−1^ is typical for materials with higher ID, such as {[Gd(HCOO)(BDC)]}_n_^36^ or {[Gd(PDA)(OX)_0.5_(H_2_O)_2_]}_n_^38^.

**Table 1:** Selected gadolinium 3D polymeric frameworks build from carboxylic acids with corresponding magnetic entropy changes (−ΔS_M_) measured at different magnetic fields and T = 1.8 K.

Compound	Linker	ID	d/Å	M/T	−ΔS_M_/J.kg^−1^.K^−1^	Ref.
MOF-76(Gd)-DMF {[Gd(BTC)(DMF)]}_n_	trimesate (BTC)	1D	4.718	5	31	[this work]
MOF-76(Gd) {[Gd(BTC)]}_n_	trimesate (BTC)	1D	4.296	5	42	[this work]
MOF-76(Gd)-H_2_O {[Gd(BTC)(H_2_O)]·4H_2_O}_n_	trimesate (BTC)	1D	4.326	5	38	[this work]
{[Gd(FOR)(BDC)]}_n_	formate (FOR) terephthalate (BDC)	2D	3.957	5	42	^36^
				3	34	
{[Gd(FOR)_3_]}_n_	formate (FOR)	3D	3.987	7	56	^37^
				3	50	
{[Gd(PDA)(OX)_0.5_(H_2_O)_2_]}_n_	oxalate (OX) propandionate (PDA)	2D	4.588	5	44	^38^
{[[Gd_2_(FUM)_3_(H_2_O)_4_]·3H_2_O}_n_	fumarate (FUM)	0D	4.588	5	21	^43^
{[Gd_2_(N-BDC)_3_(DMF)_4_]}_n_	2-aminoterephthalate (N-BDC)	0D	4.120 (dimer)	7	29	^44^
{[Gd_2_(IDA)_3_]·2H_2_O}_n_	iminodiacetate (IDA)	2D	3.840	5	37	^45, 46^

ID – ion dimensionality – propagation of gadolinium (III) ions only via carboxylate group.

(COO^−^), d – the shortest Gd···Gd distance.

For further investigation of magnetic properties, the Arrott plots in the vicinity of critical temperature for compounds **1**–3 were drawn and are shown in Fig. S7 in ESI. According to the Banerjee criterion, the positive slope of μ_0_H/M vs. M^2^ indicates a presence of second order magnetic phase transition^39,40^. The parameters A(T) and B(T) obtained from the linear region of the Arrott plots are shown in Fig. S8 in ESI. As is obvious from Fig. S7, there is no change in the slope of Arrott curves, thus we can conclude that there is no second-order phase transition present in all studied system in the examined temperature interval (1.8–30 K).

**Figure 4:**
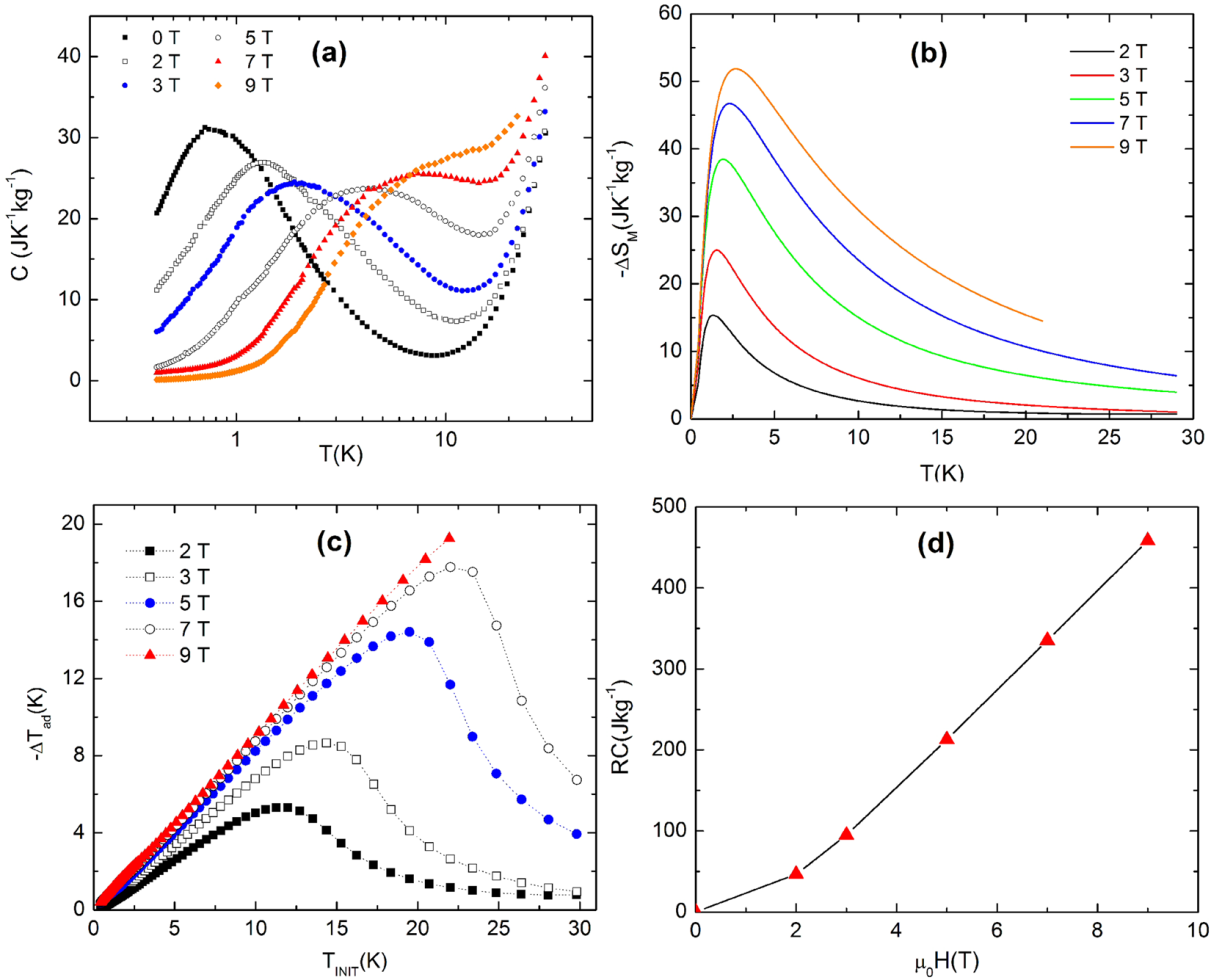
Magnetocaloric response in compound **2** studied via heat capacity measurements at temperatures 0.4 K - 30 K: (**a**) heat capacity in constant magnetic fields of 0, 2, 3, 5, 7, 9 T, (**b**) the magnetic entropy change calculated from the heat capacity data after substraction of lattice contribution, (**c**) adiabatic temperature change as a function of temperature at various changes of applied magnetic field, (**d**) calculated parameter relative cooling power RCP.

To study and confirm the magnetocaloric effect by another way the specific heat capacity measurements were realized. We have investigated the compound **2**, the sample with largest −ΔS_M_, by the temperature-dependent heat capacities (C) measurements at different external magnetic fields (0–9 T), see Fig. 4a. From the obtained C values it followed that the magnetic contribution dominated at lower temperatures (see Fig. 4a). At higher temperatures, the non-magnetic contributions due to thermal vibrations of lattice prevailed. The value of magnetic entropy change ΔS of the studied compound **2**, see Fig. 4b, was obtained from the heat capacity data by equation S_M_ = ∫ C_M_/T dT, where the magnetic heat capacity C_M_ was obtained by subtracting the lattice contribution from the total specific heat determined experimentally. The obtained value of magnetic entropy 39.5 J kg^−1^ K^−1^ at 1.8 K for magnetic field change of 5 T is in good agreement with magnetization measurements, derived from the magnetization data (42 J kg^−1^ K^−1^ at 1.8 K). The other magnetocaloric parameter, the adiabatic temperature change, −ΔT_ad_ (see Fig. 4c), was estimated as a function of the initial temperature during the adiabatic change from non-zero initial magnetic field, to zero magnetic field^41^. As it is demonstrated on Fig. 4c, the −ΔT_ad_ *vs*. initial temperature dependences are characterized by a maximum, which is shifted to higher temperatures in higher magnetic fields. For example, the maximal values of adiabatic temperature change −ΔT_ad_ at the proximity of T_init_ ∼ 23 K increase from −ΔT_ad_ = 11 K for ΔH = 1 T to −ΔT_ad_ = 18 K for ΔH = 7 T. This demonstrates the excellent properties of the studied system **2** with regard to its use as a potential refrigerant for magnetic cooling at low temperatures. Also, it is necessary to note, that from experimental study is evident that in studied system no first order phase transition was observed and therefore only magnetic subsystem is responsible for existence of magnetic entropy change.

Suitable parameter for the characterization of the MCE is the relative cooling power (RCP), and it can be estimated from temperature dependence of magnetic entropy change^42^. RCP is defined as: $RCP(\Delta H)={\int }_{{T}_{cold}}^{{T}_{hot}}-\,\Delta {S}_{M}(T,\,\Delta H)dT,$ where ΔH is the difference between the applied fields, T_cold_ and T_hot_ are the temperatures of reservoirs, −ΔS_M_ is magnetic entropy change. RCP parameter is thus the measure of the energy that can be transferred between the hot and cold reservoirs. The calculated RCP values for compound **2**, with highest value of magnetic entropy change, have been found in the range of 50 J kg^−1^–460 J kg^−1^ with increasing magnetic field from 2 T–9 T, see Fig. 4d.

## Conclusions

The effect of presence/absence of solvent molecules in channel system of MOF-76(Gd) on structural and magnetic properties was investigated. Magnetic studies suggest the presence of ferromagnetic couplings between the intrachain Gd(III) ions and large magnetocaloric effects (MCEs) with −Δ*S*_max_ = 32 J.kg^−1^.K^−1^ (MOF-76(Gd)-DMF), 41 J.kg^−1^.K^−1^ (MOF-76(Gd)) and 38 J.kg^−1^.K^−1^ (MOF-76(Gd)-H_2_O) under 5 T applied field. Observed results could be attributed to different distances between Gd···Gd ions in the framework of the samples: 4.718 Å for MOF-76(Gd)-DMF, 4.296 Å for MOF-76(Gd) and 4.326 Å for MOF-76(Gd)-H_2_O caused by solvent exchange effect. Heat capacity measurements confirmed the value of magnetic entropy S obtained from the magnetization data. The studied three metal-organic frameworks can be considered as an attractive candidate as cryogenic magnetorefrigerants.

## Supplementary information

Supplementary Table 1-3
