# Supplementary material for: Large and tunable magnetocaloric effect in gadolinium-organic framework: tuning by solvent exchange
Source: Sci Rep. 2019 Oct 30;9:15572. doi: 10.1038/s41598-019-51590-2 (PMC6821888; doi:10.1038/s41598-019-51590-2)
Supplement: Supplementary file 1 — Supplementary Table 1-3 [file 41598_2019_51590_MOESM1_ESM.pdf]

SUPPORTING INFORMATION

Large and tunable magnetocaloric effect in gadolinium-organic framework: tuning by solvent exchange

Vladimír Zeleňák[a]*, Miroslav Almáši[a], Adriána Zeleňáková[b], Pavol Hrubovčák[b], Róbert Tarasenko[b], Sandrine Bourelly[c], Philip Llewellyn[c]

[a] *Institute of Chemistry, Faculty of Science, P.J. Šafárik University in Košice, Moyzesova 11, SK-041 54 Košice, Slovakia*

[b] *Institute of Physics, P.J. Šafárik University, Park Angelinum 9, 04001 Košice, Slovakia*

[c] *Aix-Marseille University, CNRS, MADIREL, F-13397 Marseille 20, France*

**Corresponding author: vladimir.zelenak@upjs.sk*

Figures and tables

Table S1 Thermal analysis data including the released species, mass loss temperature range and enthalpic effect.

Compound	Compo-nent	Weight calcd. / wt.%	Weight obs. / wt.%	Temp. range / °C	Enthaplic effect temp. / °C
MOF-76(Gd)-DMF	H_2O, DMF	20.00	20.89	80-400	endo/ 135, 158
	BTC	40.18	39.44	400-900	exo/526 endo/646
	Gd_2O_3	40.87	39.54		
MOF-76(Gd)	BTC	50.22	50.68	400-900	exo/540 endo/650
	Gd_2O_3	49.78	49.32		
MOF-76(Gd)-H_2O	H_2O	19.82	20.18	80-400	endo/ 146, 167
	BTC	40.27	39.67	400-900	exo/527 endo/642
	Gd_2O_3	40.97	40.75		

temp – temperature, clcd – calculated, obs – observed, endo – endothermic, exo – exothermic

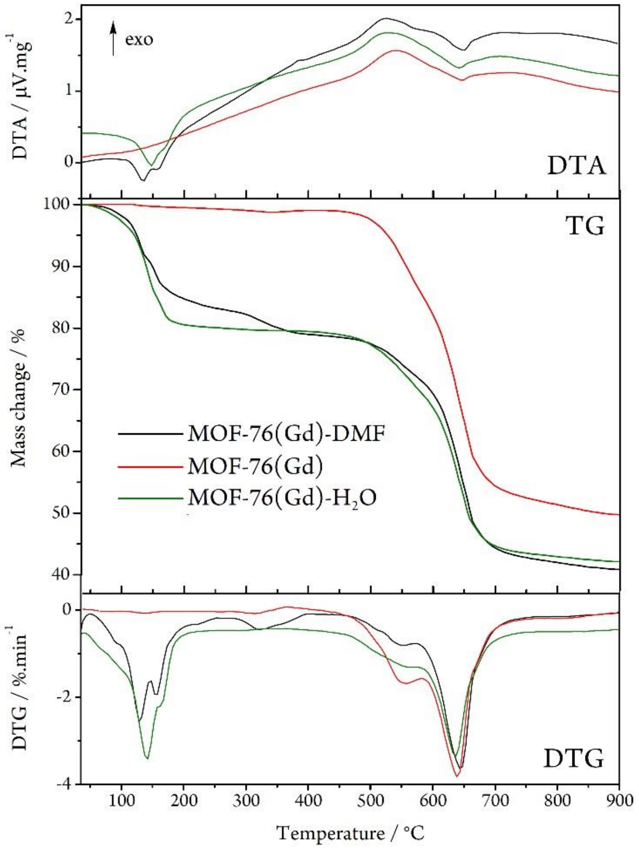

Fig. S1 TG/DTG-DTA curves of the samples measured in temperature range 40-900°C in air.

Table S2 Assignment of characteristic absorption bands in the FT-IR spectra for the building blocks in prepared samples.

Building block	MOF-76(Gd)-DMF (**1**)	MOF-76(Gd) (**2**)	MOF-76(Gd)-H_2O (**3**)
H_2O			
υ(OH)	3410(m, br)	-	3393(s, br)
DMF			
$\upsilon(CH)_{aliph}$	2955(w) 2927(w)	-	-
υ(C=O)	1667(m)	-	-
BTC			
$\upsilon(CH)_{ar}$	3098(w) 3068(w)	3070(w)	3094(w) 3070(w)
$\upsilon(COO)_{asym}$	1614(s)	1612(s)	1614(s)
$\upsilon(CCH)_{ar}$	1574(m) 1534(m)	1550(s)	1560(s)
$\upsilon(COO)_{sym}$	1440(s) 1382(s)	1439(s) 1377(s)	1437(s) 1375(s)
δ(COO)	772(m)	761(m)	763(m)

υ – valence vibration, δ – deformation vibration, ar – aromatic, aliph – aliphatic, sym – symmetric, asym – asymmetric, s – strong, m – medium, w – weak, br – broad

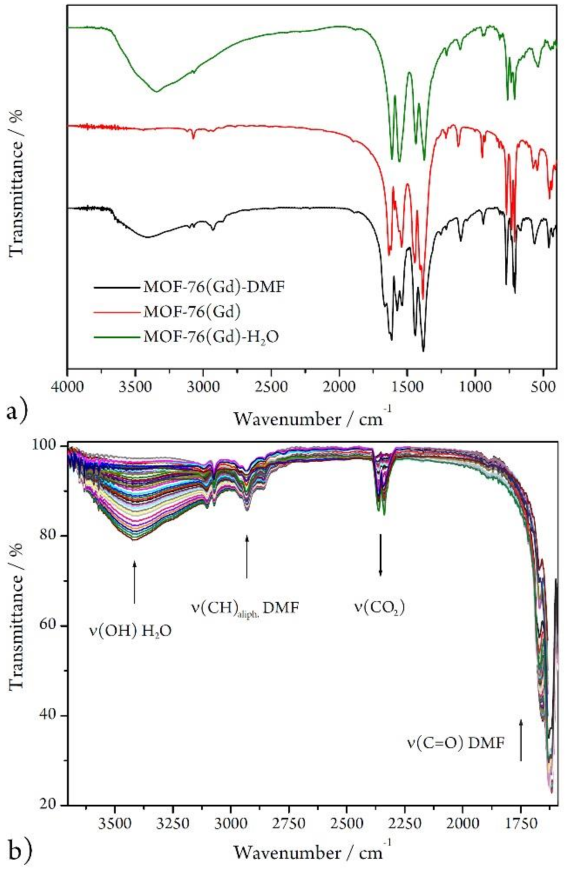

Fig. S2 a) FT-IR spectra of the prepared samples. b.) FT-IR spectra measured during heating of the compound MOF-76(Gd)-DMF in the temperature range 20-400°C.

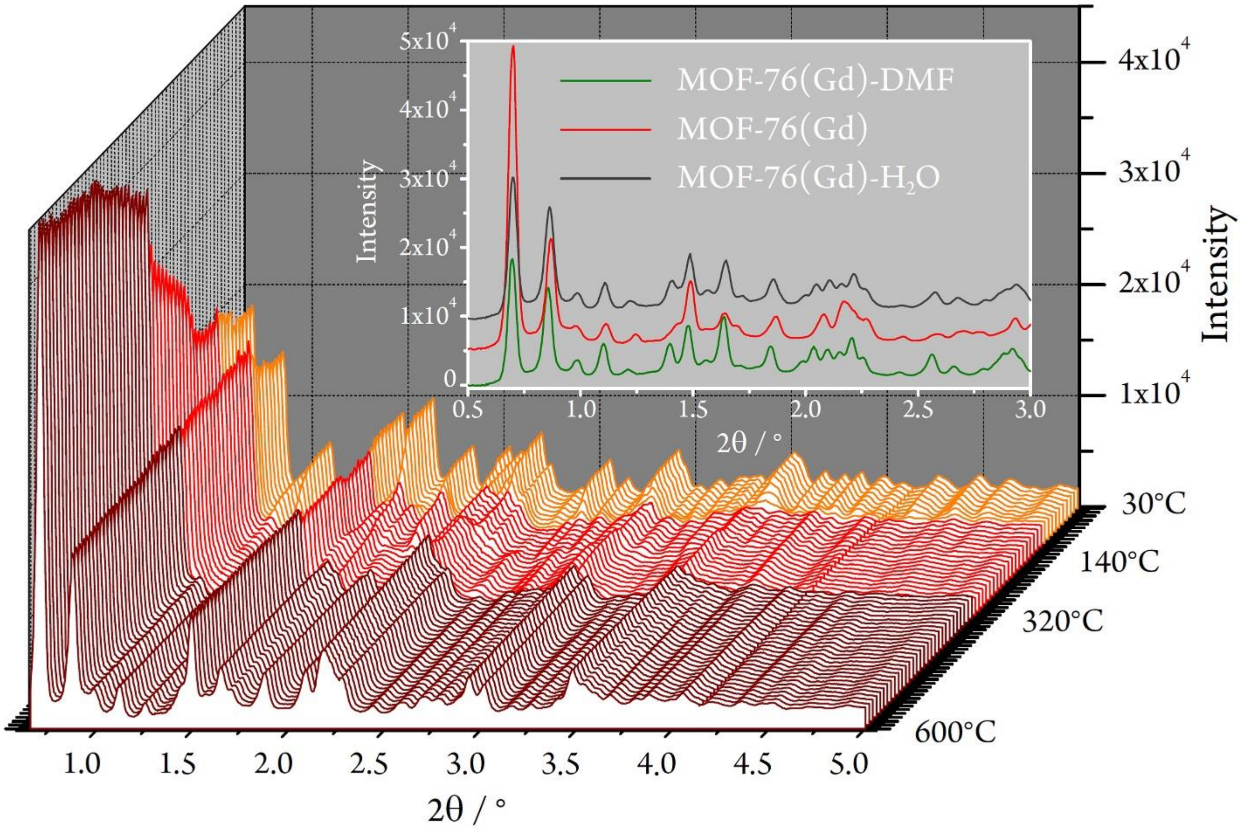

Fig. S3 HEPXRD patterns of MOF-76(Gd)-DMF measured during *in-situ* heating in the temperature range 25-600°C in air. Inset shows the diffraction patterns of all three studied samples.

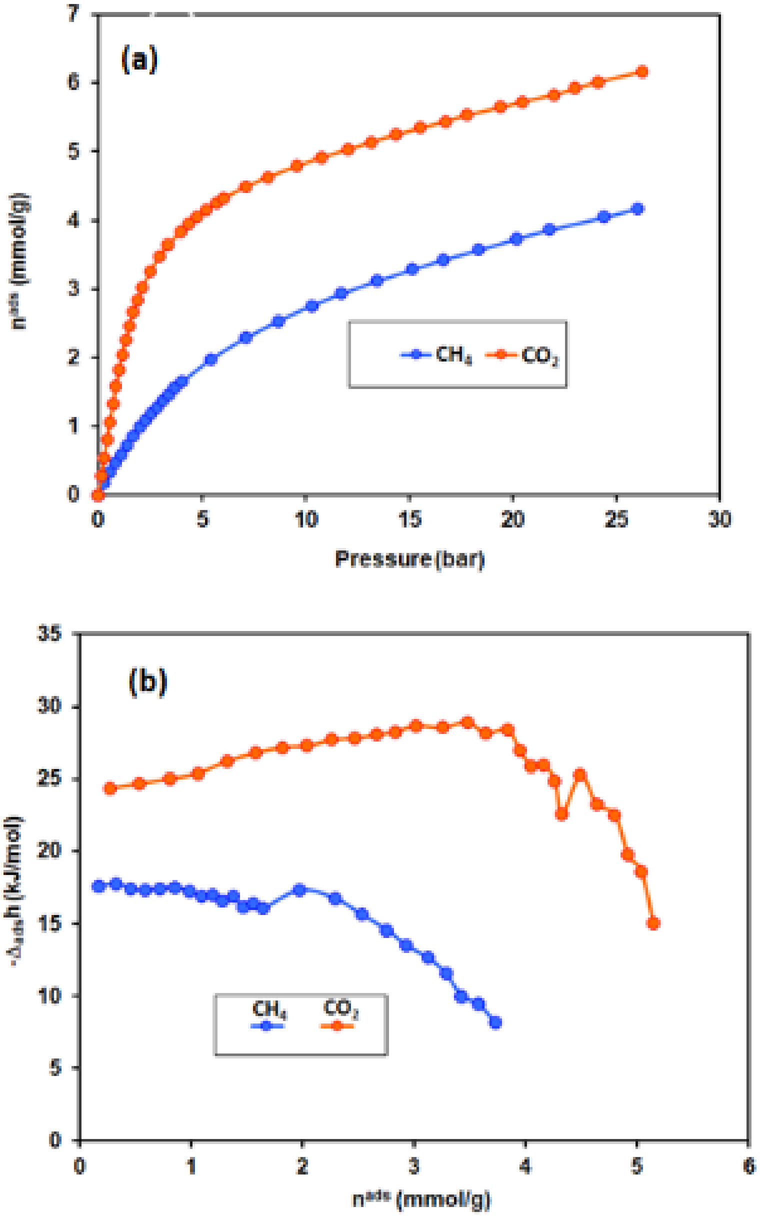

Fig. S4 a.) Adsorption of methane and carbon dioxide on MOF-76(Gd) (sample 2) at 303 K; b.) Adsorption enthalpies of methane and carbon dioxide on on MOF-76(Gd) (sample 2) measured at 303 K.

Magnetocaloric properties: Magnetocaloric effect was studied using SQUID based magnetometer MPMS 5XL up to applied field of 5 T. The relationship between changes in magnetization and magnetic entropy was expressed for an isothermal-isobaric process as

$$\Delta S_M = \int \left(\frac{\partial M}{\partial T} \right)_H dH \qquad \text{(S1)}$$

Isothermal magnetization curves were obtained following standard protocol for M(H) data collection [46]. The sample was cooled down in zero magnetic field to 30.8 K and subsequently isothermal magnetization curves M(H) were recorded in a temperature range from 20.8 to 1.8 K with a step of 0.5 K in applied fields up to 5 T.

For the discrete measurements an approximation of the equation (S1) was used

$$\Delta S_M \left(\frac{T_{n+1}+T_n}{2}, H \right) = \sum \frac{(M_{n+1}-M_n)_H}{T_{n+1}-T_n} \Delta H \qquad \text{(S2)}$$

where M_n and M_{n+1} are the magnetization values measured in magnetic field H at temperatures T_n and T_{n+1} , respectively.

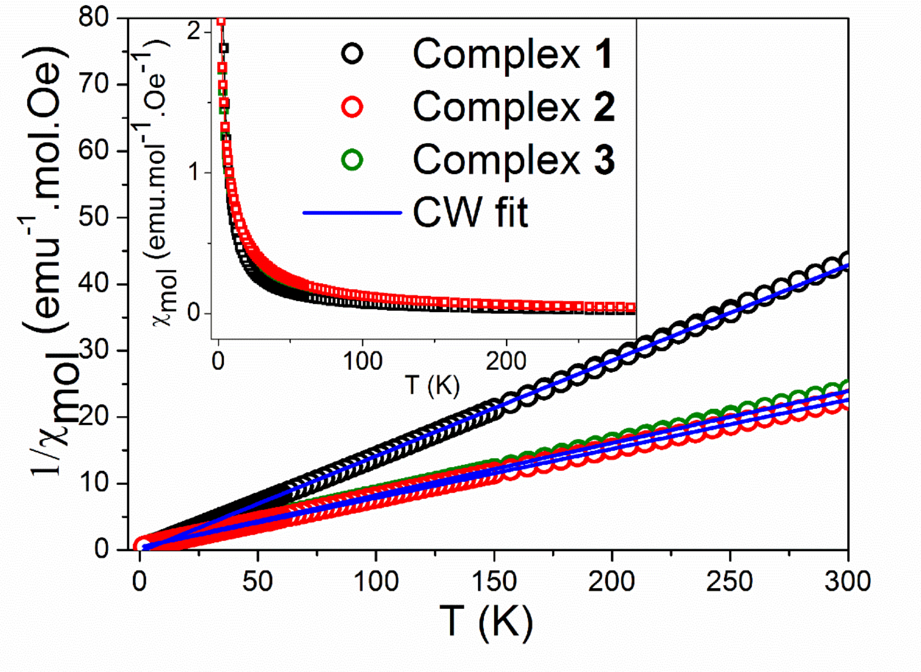

Fig. S5 The reciprocal value of molar magnetic susceptibility with a linear fit according to the Curie–Weiss law. Inset shows the temperature dependence of molar magnetic susceptibility.

Table S3 Magnetic parameters.

**Compound**	**Θ/ K**	**J/k_B/ K**	**μ_{eff}**
MOF-76(Gd)-DMF	1.54	0.3	7.74
MOF-76(Gd)	-6.23	-1.8	10.3
MOF-76(Gd)-H_2O	-5.58	-1.06	9.9

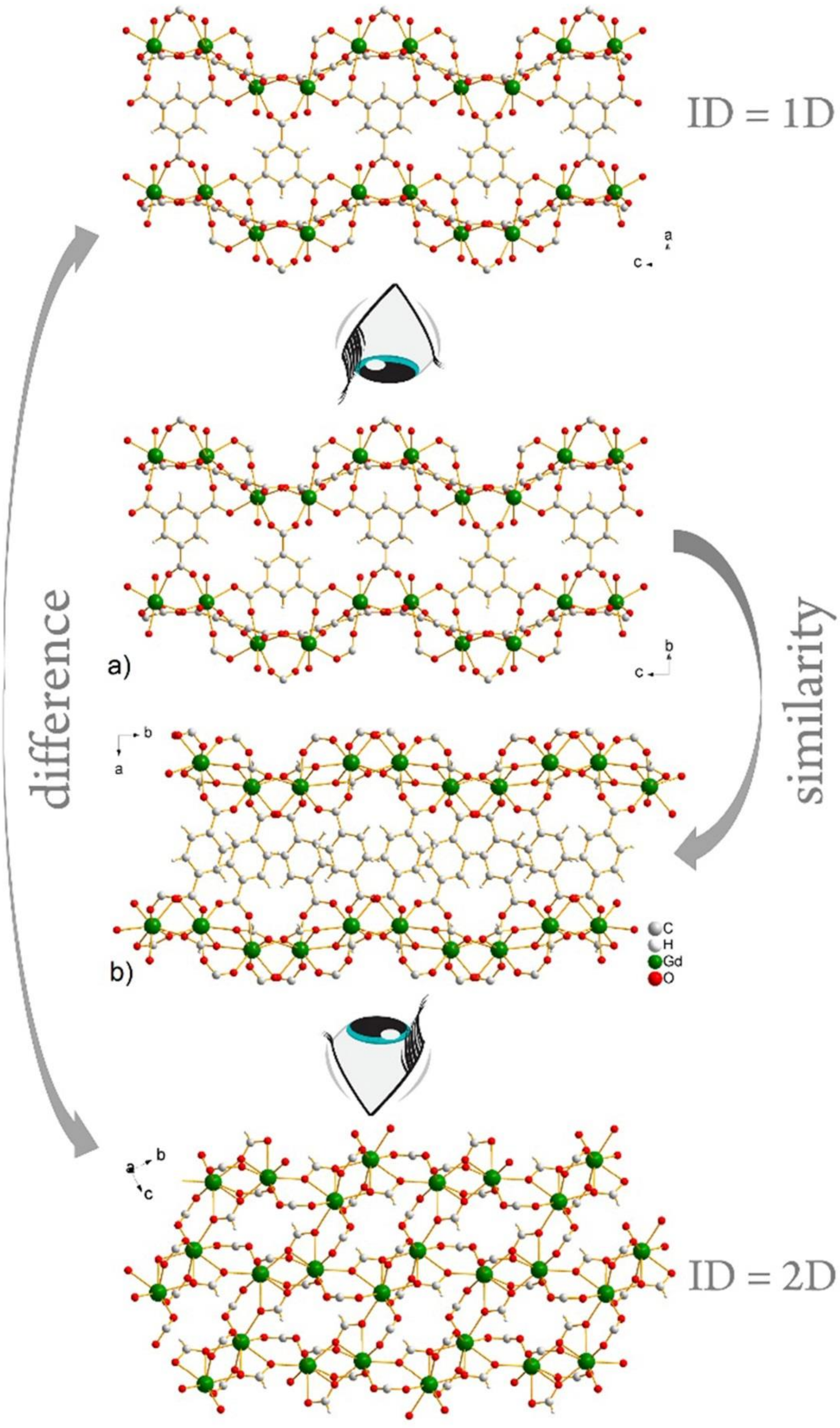

Fig. S6 A view of the similarity/difference between the crystal structure of a) MOF-76(Gd) and b) compound $\{[Gd(HCOO)(BDC)]\}_n$ with corresponding ion dimensionality.

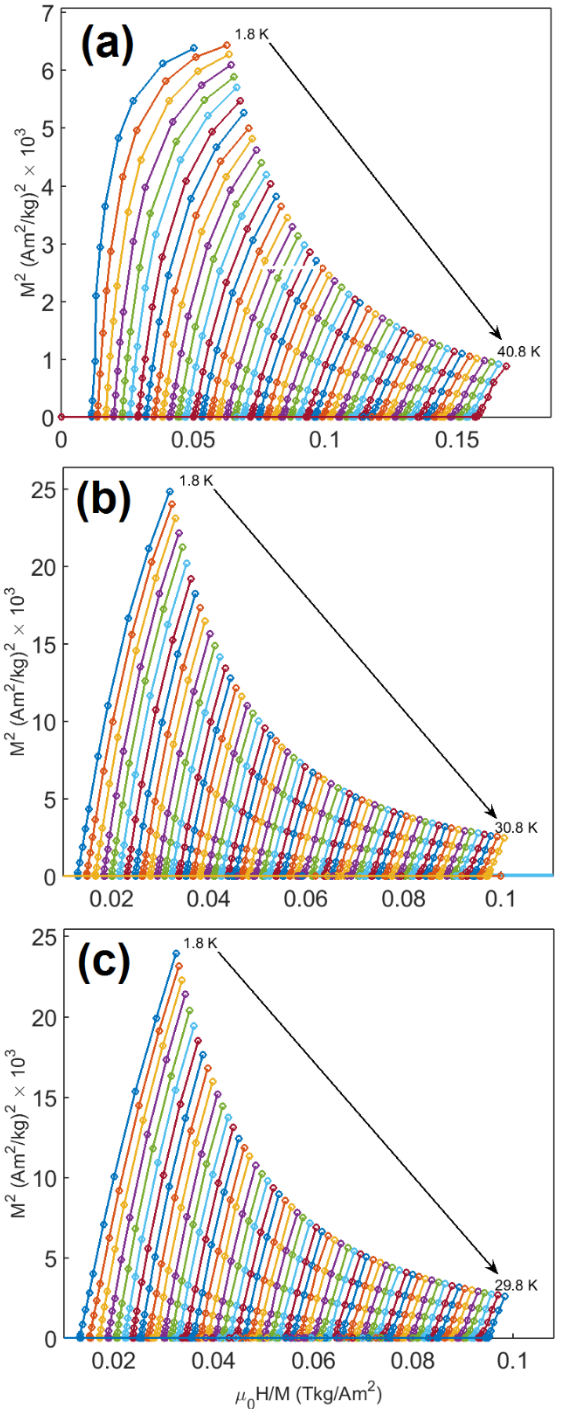

Fig. S7 Arrot plots of studied compounds up to applied field of 5 T calculated from isothermal magnetization data. **1** (a), **2** (b) and **3** (c).

According to Landau theory, a theoretical modeling of the MCE could also provide the proof of characteristics typical of magnetic transition. The Gibbs free energy can be expressed as:

$$G(T,M) = G_0 + \frac{1}{2}A(T)M^2 + \frac{1}{4}B(T)M^4 - \mu_0 HM, \quad \text{(S4)}$$

where A(T) and B(T) parameters are temperature dependent representing the electron condensation energy and magnetoelastic coupling. Assuming the condition of equilibrium we obtain

$$\frac{\mu_0 H}{M} = A(T) + B(T)M^2 \quad \text{(S5)}$$

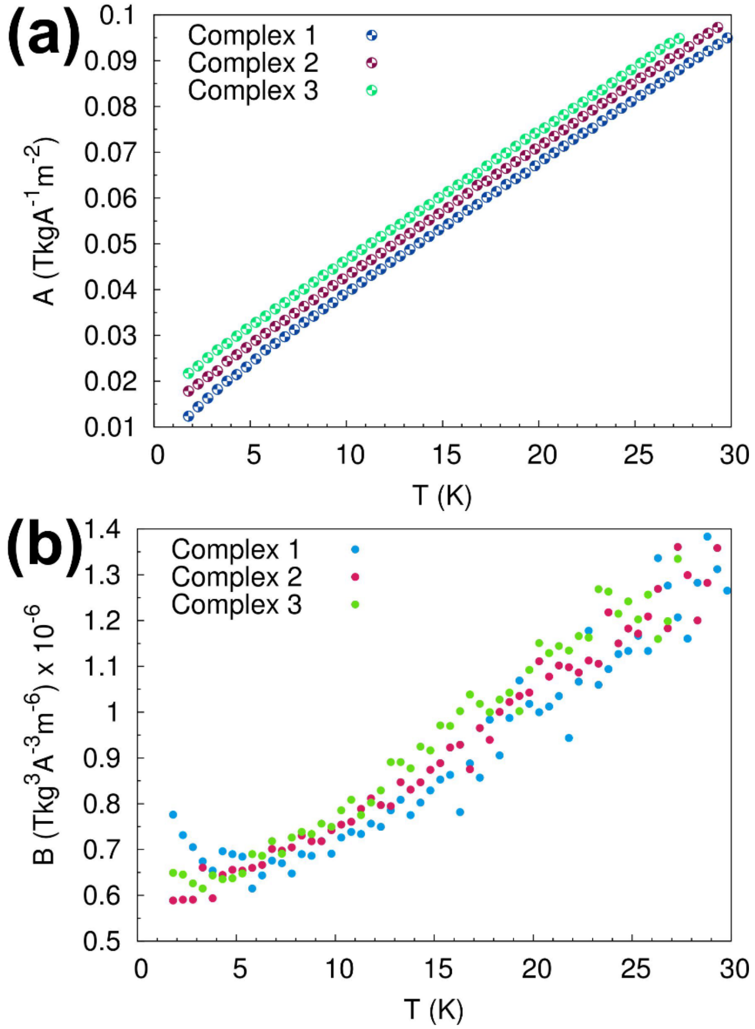

Fig. S8 Temperature dependence of the parameters A(T) and B(T) of studied complexes.
